# Energy Performance Assessment of Innovative Building Solutions Coming from Construction and Demolition Waste Materials

**DOI:** 10.3390/ma14051226

**Published:** 2021-03-05

**Authors:** Beatriz Fraga-De Cal, Antonio Garrido-Marijuan, Olaia Eguiarte, Beñat Arregi, Ander Romero-Amorrortu, Giulia Mezzasalma, Giovanni Ferrarini, Adriana Bernardi

**Affiliations:** 1 Tecnalia, Basque Research and Technology Alliance (BRTA), 48160 Derio, Spain; antonio.garridomarijuan@tecnalia.com (A.G.-M.); olaia.eguiarte@tecnalia.com (O.E.); benat.arregi@tecnalia.com (B.A.); ander.romero@tecnalia.com (A.R.-A.); 2 Enedi Research Group, Thermal Engineering Department, University of the Basque Country (UPV/EHU), 48013 Bizkaia, Spain; 3 Research and Environmental Devices (RED srl), Viale dell’Industria, 58E, 35129 Padua, Italy; giulia.mezzasalma@red-srl.com; 4 Construction Technology Institute, CNR National Research Council of Italy, Corso Stati Uniti 4, 35127 Padua, Italy; giovanni.ferrarini@itc.cnr.it; 5 National Research Council–Institute of Atmospheric Sciences and Climate (CNR-ISAC), Corso Stati Uniti 4, 35127 Padova, Italy; a.bernardi@isac.cnr.it

**Keywords:** building retrofitting, energy conservation measures, construction and demolition waste, building model calibration

## Abstract

Prefabricated solutions incorporating thermal insulation are increasingly adopted as an energy conservation measure for building renovation. The InnoWEE European project developed three technologies from Construction and Demolition Waste (CDW) materials through a manufacturing process that supports the circular economy strategy of the European Union. Two of them consisted of geopolymer panels incorporated into an External Thermal Insulation Composite System (ETICS) and a ventilated façade. This study evaluates their thermal performance by means of monitoring data from three pilot case studies in Greece, Italy, and Romania, and calibrated building simulation models enabling the reliable prediction of energy savings in different climates and use scenarios. Results showed a reduction in energy demand for all demo buildings, with annual energy savings up to 25% after placing the novel insulation solutions. However, savings are highly dependent on weather conditions since the panels affect cooling and heating loads differently. Finally, a parametric assessment is performed to assess the impact of insulation thickness through an energy performance prediction and a cash flow analysis.

## 1. Introduction 

The building sector is one of the largest consumers of resources at European level in terms of both material and energy aspects throughout all stages of a construction project. In fact, one third of the waste generated in Europe comes from Construction and Demolition Waste materials (CDW) [1]. To give a deeper insight, it is important to remark that approximately 3 billion tons of waste materials are generated in the European Union each year. Out of this, around 1 billion tons come from construction and demolition activities, with a large quantity of CDW materials ending up in landfills without any form of recovery or reuse [2]. Therefore, one of the pillars of the EU roadmap for the decarbonization of the European economy and the reduction of waste production is focused on the building sector [3].

The innovation on the end-of-life management of materials must be in line with the target of the Waste Framework Directive 2008/98/EC to be achieved by 2020, which requires 70% of CDW to be prepared for reuse, recycling, and other types of recovery [4]. The success of the existing quality assurance/control systems for the recycling of building materials (as the European Quality Association for Recycling) [5] results in a high-quality product with potential for market uptake.

Moreover, the revision of the Waste Framework Directive 2008/98/EC has consolidated the primary role of waste prevention, providing a five-step hierarchy of waste management. Besides optimizing the recyclability properties of materials, solutions for the recycling and reuse of existing building structures must be developed.

Several studies have assessed the feasibility of different construction products reusing CDW materials. Experimental results obtained by Bravo et al. [6] show that, after the incorporation of up to 25% of CDW, the variation of the properties of the concrete remains within the established limits. Panizza et al. [7] presented the mechanical and physical characterization of a geopolymer mortar embedding inorganic recycled aggregates from CDW. Additionally, experimental results by Panizza et al. [8] illustrate promising properties of geopolymers with up to 50% of CDW for use in building elements.

Further studies show the promising potential of geopolymers, which can exhibit a wide variety of properties and characteristics depending on the mixing material selection, inert and filler used, and processing conditions, including high compressive strength, low shrinkage, short setting time, and good resistance to chemicals (acids, bases, and organic solvents). They are mechanically stable up to 1000–1200 °C and are intrinsically non-flammable, providing passive fire-resistance, with good behavior towards UV, high humidity, and aggressive chemicals [9,10]. Furthermore, the intrinsic ability of geopolymer surfaces to reproduce fine details opens up for a wide variety of products allowing for architectural freedom.

Geopolymers are considered green materials for a sustainable economy [11] since they derive from natural sources allowing the reuse of industrial by-products (furnace slag, fly ash, etc.). Their ability to be prepared simply at room temperature implies 10 times less CO_2_ emissions than Portland cement, reducing the raw materials consumption by using industrial by-products. Finally, they have an easy end of life disposal and may be reused as raw materials. Geopolymers exhibit a highly improved life cycle compared to mainstream building materials such as Portland cement while keeping very good mechanical properties and excellent fire resistance. Furthermore, geopolymers also present an interesting way to recycle organic CDW such as wood. Indeed, geopolymers are an inorganic binder that can be used similarly to historically used magnesite or Portland cement binders for producing cement bonded wood wool panels and particle boards [12].

A second way to completely recycle CDW using concrete is re-clinkering the hydrated cement using standard cement kiln procedures. However, this process consumes a significant amount of energy and releases large quantities of CO_2_.

Despite promising research results, the widespread adoption of CDW in construction projects remains a challenge due to the lack of confidence among stakeholders [13]. Despite the high potential for the incorporation of circular economy practices, there is still an extent of uncertainty in the quality of CDW recycled materials due to inadequate information and negative perception associated with these products and unexpectedly high cost. This is especially relevant when considering the energy performance of buildings, since involved technicians have little reliable information on the influence of these products on the energy demand [14,15].

This issue is not trivial, as the building sector concentrates 40% of the final energy use in Europe [16]. The energy efficiency of buildings is one of the key priorities of the EU, as reflected in the Energy Efficiency Directive [17], which poses a special focus on supporting environmentally sound decisions with energy efficient solutions. Building energy performance is largely dependent on the materials used [18], and thus there is a need for advanced and high-performance construction materials. In order to increase the energy performance of existing buildings, External Thermal Insulation Composite systems (ETICS) and ventilated façade systems are commonly used because of their proven effectiveness for improving thermal performance [19,20,21]. It is also necessary to improve the understanding of material and component behavior in the whole life cycle to contribute to lower embodied energy and resource efficiency. For these reasons, further actions are necessary to lower the content of embodied energy of building elements. Therefore, new materials combining structural properties, thermal resistance, and/or light weight need to be developed [22].

Even though the materials of the envelope represent a key factor in the overall energy efficiency of a building, only a limited number of studies have considered the combination of energy efficiency and circular economy to develop energy efficient solutions and assess the environmental performance of façade systems or similar products [23,24,25]. Hence, further research is needed in order to develop innovative materials and solutions that not only reduce the energy used on the production process, but also decrease the energy demand of the buildings.

InnoWEE (INNOvative prefabricated components including different Waste construction materials reducing building Energy and minimizing Environmental impacts) [26] is a research project developing innovative solutions from recycling CDW. The overall objective of the project is to use geopolymer technology to produce a panel-based system incorporating high amounts of CDW. These panels are used as insulating solutions in the renovation of external walls of buildings. Two prefabricated innovative solutions have been developed and tested under real conditions within the InnoWEE project in three demo sites located in different climate conditions: Voula (Greece), Padova (Italy), and Bucharest (Romania). Further details about the project developments can be found in Fodor et al. [27] and Kvočka et al. [28]. The thermal performance of the InnoWEE products has been analyzed for the different demo sites through exhaustive modelling and simulations to assist with the calculations of building energy savings. The models have been calibrated and adjusted with real measured data according to the International Performance Measurement and Verification Protocol (IPMVP). Furthermore, a cost-efficiency analysis for different thicknesses of insulation has been performed to optimize and support the selection of the most suitable solutions. The overall objective is not only to assess the thermal performance of the panels, but also to study the impact of insulation thickness on the energy savings.

## 2. Methodology

### 2.1. Description of InnoWEE Solutions

This section describes the ETICS-like panels and the ventilated façade developed within the InnoWEE project. As described by Kvočka et al. [28], two types of geopolymer mixtures have been developed to manufacture these solutions: (i) High-density geopolymer (HDG), which embeds approximately 50% by weight of inorganic CDW aggregates (from fired clay, mortar, and concrete rubble); and (ii) wood geopolymer (WG), which incorporates at least 40% of CDW wood particles. In regard to raw material information and mixing methodology, either potassium silicate (HDG) or sodium silicate (WG) were part of the liquid mixtures. Aggregates for those HDG solutions came from inorganic harmless CDW materials, while softwood waste was used for WG production.

Geopolymers are inorganic polymers obtained from the reaction of an aluminosilicate powder with an aqueous alkaline solution. The HDG material was developed using geopolymeric technology to produce a system of panels incorporating high amounts of CDW. The geopolymer binder was based on commercial solid precursors such as metakaolin, ground granulated blast furnace slag, and class F fly ash.

Regarding the wood geopolymer, it is a similar material to a cement-bonded particleboard, but with the wood aggregates kept together by a geopolymer binder able to set at ambient temperature. Therefore, the wood geopolymer panel embeds 40% by weight of wood particles, resulting in an apparent density of about 1.0 g/cm^3^ in dry conditions and comprised between 1.1 and 1.2 g/cm^3^ in environmental conditions (interior ambient).

#### 2.1.1. ETICS-Like Panels

ETICS-like insulation panels have surface dimensions of 40 × 90 cm, with an inner layer of expanded polystyrene (EPS) and an outer layer of HDG coming from CDW, with the dimensions in Table 1 below. The physical properties of ETICS-like panels were experimentally measured as per EN 12667:2001 [29].

#### 2.1.2. Ventilated Façade

The ventilated façade cladding panels are made from geopolymer technology with surface dimensions of 59.5 cm × 59.5 cm. Panels are composed of an outer HDG layer (8 mm thick) with two horizontal ribs, and 7 mm thick WG inserts. Cladding panels are supported at four points using adjustable stainless-steel body anchors, allowing a 50 mm thick rear-ventilated air cavity behind the panels. The cladding panels are installed with a 5 mm wide open joint between them, both horizontally and vertically. Therefore, the cavity is ventilated all along its height, and not only through the lower and upper parts. The arrangement of the cladding panels and the fixing system are described in detail in [30].

If there was thermal insulation between the existing wall and the ventilated cavity, as is typical for such ventilated façade systems, the metallic support elements would break through that thermal insulation. A desktop assessment using three-dimensional numerical modelling was performed for estimating the impact of these thermal bridges on the heat flow and potential associated moisture risks [30]. The study concluded that the risk of condensation and mold growth on internal surfaces is successfully prevented by thermal insulation, but the multi-dimensional heat flow associated with the anchors grows as insulation levels increase. However, if no gaps are present in the insulation in the area around the anchors, the additional heat flow associated with the support elements keeps in a range of 8–13%, showing better thermal performance than typical aluminum-based systems comprising vertical profiles supported by brackets.

### 2.2. Energy Performance Assessment

For the achievement of a successful Building Energy Model (BEM), it is crucial to minimize the discrepancies between the actual measured performance and predictions from the building model. Best practices for measurement and validation are applied in this work to avoid this divergence known as “performance gap” [31]. Equivalent building models bring a precise resemblance between the design and the operational building.

Complete software building models allow for the evaluation of the energy demand under different conditions. DesignBuilder [32] building modelling software (v6.01, DesignBuilder Software Ltd Stroud, Gloucestershire, UK) based on the open-source Energy Plus simulation engine (v8.9, U.S. Department of Energy’s (DOE), Building Technologies Office (BTO) and NREL, Golden, CO, USA) has been used, allowing the modelling of complex geometries and the performance evaluation of alternative construction designs. In order to estimate the energy savings achieved by InnoWEE solutions, the energy performance of the existing building (baseline period) is compared with the retrofitted scenario (reporting period). The energy assessment has been performed in accordance with the principles and best practices in International Performance Measurement and Verification Protocol (IPMVP) [33]. Option D (calibrated simulation) has been used to conduct the study, calculating energy savings through the simulation of energy consumption and demand of either the whole facility, or sub-facility. The energy outputs of the model have been calibrated using hourly measured data or monthly billing data.

Firstly, the building is modelled in its existing condition and simulated for a baseline period (scenario 0) with EnergyPlus. The thermal behavior of every zone is calculated based on the building features, HVAC schedules, occupancy patterns, internal gains, and system loads. The process meets EN ISO 15927 1-6 [34], EN ISO 52000-1 [35], ASHRAE Handbook of Fundamentals (2005) [36], and EN ISO 13790:2008 [37].

Following this, Heating Degree Days (HDD) and Cooling Degree Days (CDD) [38] are applied in order to weather-normalize energy consumption between the building model and real data as described in Equation (1). This normalization allows a like-for-like comparison between the outdoor conditions of a typical year used in the building simulation, and real measured outdoor data. Typical Meteorological Years (TMY) are derived from hourly observations at a specific location by the national weather service or meteorological office. They are data sets that typify one year of hourly climate data extracted from at least 10‑year records [39]. The climate datasets in the present study are based on recorded observations and predictions for future scenarios induced by climate change have not been considered.

(1) $$Q_{\mathrm{met},\mathrm{norm}}=Q_{met\cdot }\frac{HDD_{mod}}{HDD_{real}}\;\left[kWh\right]$$

where $Q_{\mathrm{met},\mathrm{norm}}$ is the normalised energy data [kWh], $Q_{met}$ is the metered energy data [kWh], $HDD_{mod}$ is the number of heating degree days of the TMY of the model, HD is the number of heating degree days for the measured conditions. An analogous equation is used for CDD.

Whenever measured weather data is available for the monitored periods, the TMY climate file is modified to include recorded outdoor temperature, solar radiation, and relative humidity.

The measured data is included in all models. The thermal characterization of the ventilated façade is based on the normalized procedure described in ISO 6946:2017 [40]. The methodology discards the thermal resistance of the air layer and the cladding and considers a surface resistance of 0.13 m^2^·K/W on the former external surface wall.

**Figure 1:**
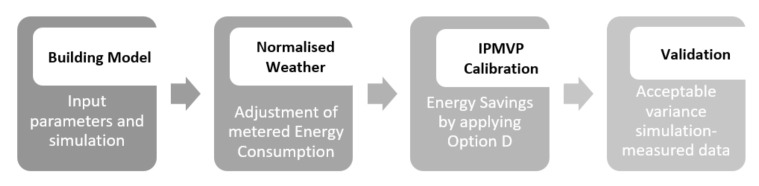
Outline of the model calibration process.

Later, calibration data is modified to meet an acceptable level of fluctuation between simulation results and monthly utility bills. Operating and influential parameters such as occupancy profiles, internal gains, equipment, and ventilation rates are commonly included in this process. Then, InnoWEE solutions are applied to the calibrated model to build the reporting period (scenario 1) and obtain the energy consumption after retrofitting measures. Finally, normalized savings are determined with the calibrated simulation results representing the baseline period (before retrofitting) and the reporting period (after retrofitting). Additionally, the impact of the insulation thickness is assessed on the building energy needs for all demo sites by means of parametric simulations. Figure 1 shows the outline of the model calibration process.

### 2.3. Optimization

Once the simulation models are developed and validated, the energy demand of the building can be predicted under different scenarios to optimize the solutions with the best compromise between investment, operation cost, and energy efficiency. This parametric optimization focuses on economic Key Performance Indicators (KPIs) to understand the investment needed and the savings on the energy costs after the building renovation. The economic KPIs are:

(a) Annual economic savings after retrofit.
(b) Payback time required for the return of an investment.

In energy-efficient renovation projects, the initial investment is eventually paid off by the avoided operational cost associated with energy savings. The cumulative cashflow is calculated as per Equation (2).

(2) $$C_{flow}=\sum_{n}^{i}\left[Q_{annual}\cdot C_{elect}\right]-C_{inv}$$

where $C_{flow}$ is the cumulative cashflow in year n [€], n is the number of years considered in the calculation [a], $Q_{annual}$ is the annual energy saving from the retrofit measures [kWh/a], $C_{elect}$ is the cost of electricity for year i [€/kWh], and $C_{inv}$ is the investment cost of the retrofit measures [€].

The optimization is performed based on a sensitivity analysis on the economic KPIs. KPIs for a range of insulation thicknesses are presented in the results and discussion section.

## 3. Demo Cases and Model Validation

Three demo sites of the InnoWEE project are selected for this study, located in Romania, Italy, and Greece. All of them had parts retrofitted with both the ETICS-like panels and the ventilated façade. The present section focuses on the Old City Hall of Voula Municipality site in Greece, as a case study that illustrates the methodology applied to all locations.

**Figure 2:**
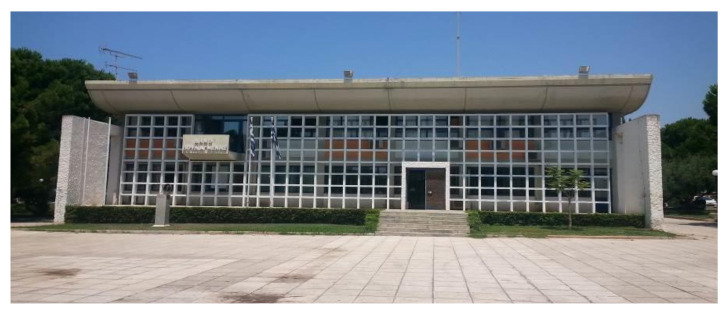
City Hall of Voula [source: InnoWEE project].

**Figure 3:**
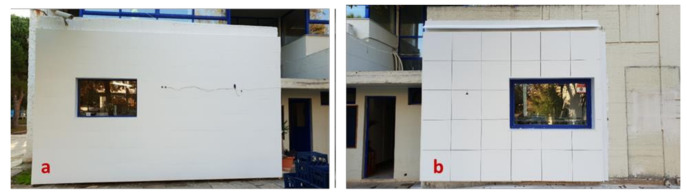
InnoWEE retrofitting: (**a**) ETICS-like panels in Office 1, (**b**) ventilated façade in Office 2 [source: InnoWEE project].

**Figure 4:**
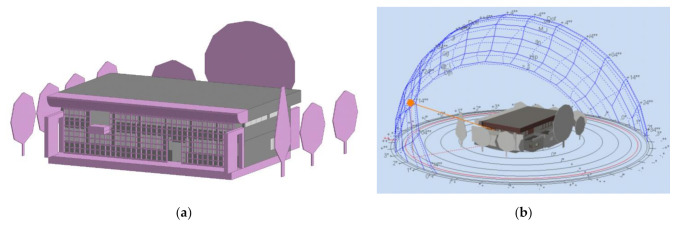
Virtual model. Voula Town Hall, Greece. (**a**) Building geometry and (**b**) Sunpath

The City Hall of Voula is a heritage building from 1967 located in a residential area in the Municipality of Voula-Vouliagmeni, Greece. It is composed of two floors and a partial basement. The main floor at ground level is an open area, while the first floor is mainly an office space with light partitions. Figure 2 shows the former state of the building before the retrofit. As depicted in Figure 3, both ETICS-like panels and the ventilated façade were installed in two different rooms on the south facing façade, Office 1 and Office 2. Figure 4 displays the 3D model of the building, including the sun path and the relevant shading components.

The existing HVAC system consists of split air conditioning units of different capacities. As the whole building has been divided in two main thermal zones, the same type of system is applied for all areas.

Regarding the validation process for this demo case, utility bills provided by the building owner were normalized by using heating and cooling degree days (see Equation (1)). Consequently, the inputs of the building model were adjusted to match simulation results with normalized metered data.

**Figure 5:**
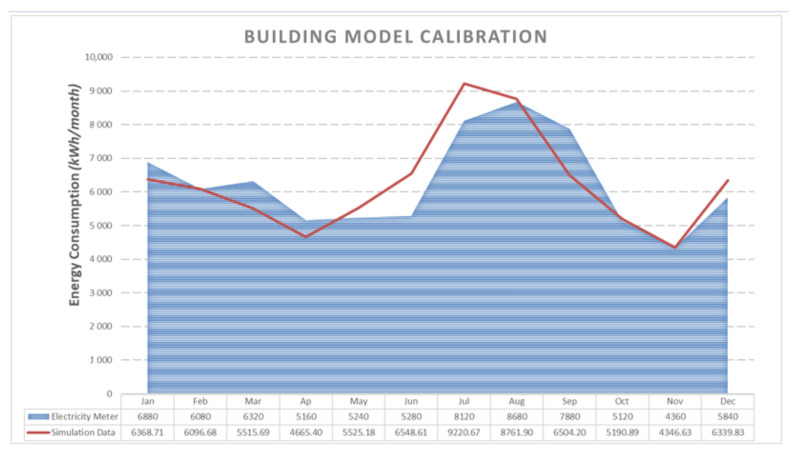
Calibration of the simulation model with utility bills for the demo building in Voula (Greece).

Figure 5 compares energy consumption results from the model with the measured energy consumption pattern. The model for the baseline (pre-retrofit) period was selected for calibration as explained in the Methodology section. The deviation between electricity bills and the building model remains within acceptable tolerances for the IPMVP protocol [33]. The model has a coefficient of determination R² = 0.86 and the coefficient of variation of the root mean square error is CV(RMSE) = 12%. Discrepancies in monthly aggregated energy consumption between model predictions and measured values range between ±0.3% and ±24%. The annual mean normalized bias error is MNBE = 0.2%.

## 4. Results and Discussion

This section presents results for the estimated energy savings, an analysis of the impact of the insulation thickness, and finally an economic assessment performed with the model previously described. A comparison of scenarios, as well as a sensitivity analysis, assesses the performance of InnoWEE solutions in the Voula demo building described in the previous section. Additionally, a summary of the results obtained in the project for all three demo sites is included, along with an environmental analysis for the operation phase focused on the reduction of the energy needs.

### 4.1. ETICS-Like Panels

Office 1 was refurbished with ETICS-like panels. The outcomes of the validated model, including former conditions (scenario 0), are compared with the outcomes coming from the simulation of the retrofitted model (scenario 1).

**Figure 6:**
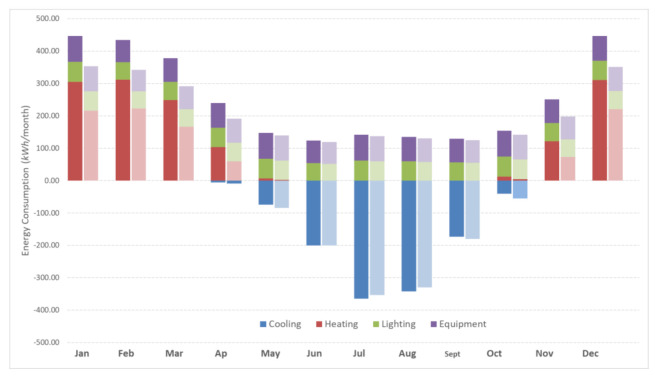
Energy needs before (scenario 0) and after (scenario 1) the placement of ETICS-like in Voula demo site.

Figure 6 illustrates the heating and cooling requirements prior to and following the façade upgrade with an insulation thickness of 7 cm. Overall, the total annual energy demand of the building is reduced considerably. However, it is worth noting that heating requirements decrease substantially, while cooling demand mainly remains steady or even increases slightly from April to October. A likely explanation is that high levels of insulation hinder the dissipation of heat gains from solar radiation, computers, or occupants. Despite that effect, the annual dynamic simulation results display total energy savings of 25.6%.

**Figure 7:**
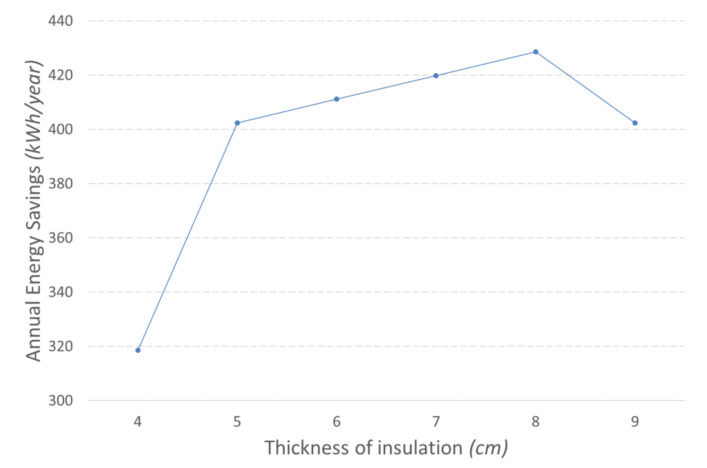
Annual energy savings against insulation thickness.

Additionally, different thicknesses of EPS are simulated to assess the impact of insulation thickness both on the building energy demand and on the return of investment of the renovation works. Figure 7 shows the result of the parametric simulation. In general, energy savings rise more sharply from 4 to 5 cm of insulation thickness. Beyond that width, the increase of insulation thickness has a lower but steady impact, gradually growing to reach the peak at 8 cm. Further increasing insulation thickness to 9 cm makes energy savings drop due to the increase on the cooling load, as explained previously.

**Figure 8:**
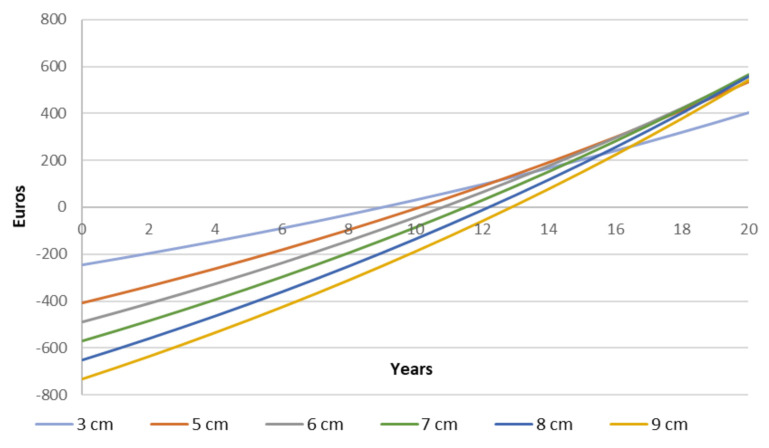
Cashflow analysis for different thicknesses of ETICS.

In terms of economic feasibility, Figure 8 shows the time required to redeem the funds expended in the investment as a function of the insulation thickness considered. It is remarkable that increasing insulation thickness also lengthens the payback period. While adding 5 cm of insulation would take close to ten years to get a return, 9 cm would take nearly 13 years. Therefore, although the highest energy savings are achieved by using an insulation thickness of 8 cm, the time required to compensate for the initial investment accounts for more than twelve years. The cumulative cashflow assessment has been performed considering the next assumptions:

(a) Cost of electricity: 0.195 €/kWh [41];
(b) annual inflation on the cost of electricity: 3%;
(c) cost of 2 cm EPS: 6.287 €/m^2^.

### 4.2. Ventilated Façade

Office 2 was refurbished with a ventilated façade as described in Section 3. A comparison of the energy consumption before (scenario 0) and after (scenario 1) the installation is performed in this section.

**Figure 9:**
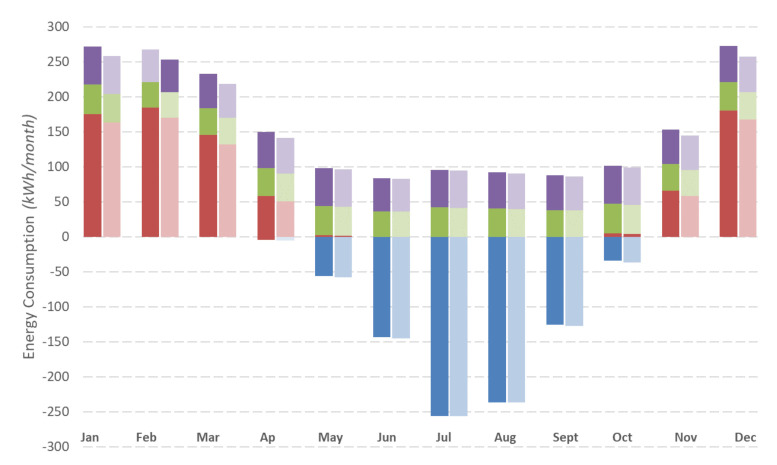
Energy needs before (scenario 0) and after (scenario 1) the ventilated façade in the Voula demo site.

Figure 9 shows a monthly comparison throughout a full year. In the same way as for ETICS-like panels, the heating energy demand of the demonstration building drops with the ventilated façade, but the cooling requirements hardly fluctuate in the summer months. The total energy saving for a full year is estimated at 2.44%.

**Figure 10:**
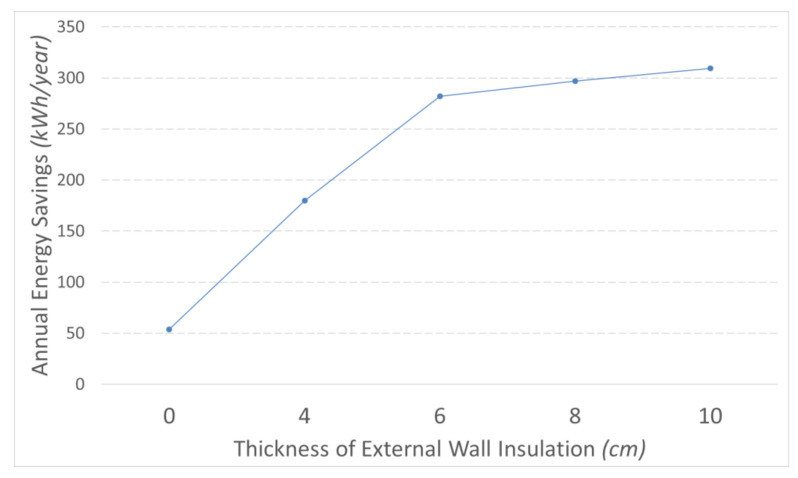
Ventilated façade including external wall insulation.

Looking into Figure 10, there is an 83% increase in the annual energy savings when 6 cm thick thermal insulation is added, reaching almost 300 kWh/year. Nevertheless, if the thickness of insulation is larger than 6 cm, energy consumption is not further reduced substantially.

Regarding the economic assessment, for the case of the ventilated façade, there is no sensible payback period since the first capital investment is considerably high when compared with the energy savings achieved. As described in [30], thermal insulation is advised along with the ventilated façade in order to avoid condensations.

From the simulation model of Voula Old Town, it can be concluded that the placement of both ETICS-like panels and ventilated façade have the potential of substantially reducing the energy consumption for Office 1 and 2, respectively.

### 4.3. Comparison of Savings among Demo Buildings

Results from the selected demonstration building, along with findings from the two other demo sites, are summarized in the present section.

Table 2 depicts the energy savings achieved using ETICS-like panels and ventilated façade in Italy, Romania, and Greece, allowing the comparison between climates, solutions, and seasons of the year.

The advantage of ETICS-like panels is apparent in Italy, with savings reaching up to 32% of total annual energy consumption. However, the reduction of savings in summer is striking. For the ventilated façade solutions, there is a dramatic fall of the total saving, accounting for nearly thirteen times lower. The seasonal pattern is similar to ETICS-like panels, with a slight rise of savings up to 1.6%.

In Greece, the behavior of the building with the novel solutions shows large similarities with the Italian demo site. The performance is more advantageous in winter than in summer. Moreover, the ETICS-like panels appear to be more suitable than the ventilated façade. Further details and analysis can be found in Section 3.

On the other hand, the models for Romania showed less savings than the other demo sites. The energy savings along the year are similar for both ETICS-like and ventilated façades, approximately 4%. The reason for the difference with the other demonstration sites might lie on the outdoor conditions. In this case, there is no significant difference attributed to the season.

All simulations and validations were based on recorded climate data and future scenarios due to climate change have been left out of the scope of the present study. Further work might focus on the potential impact of extreme conditions posed by cooler winters, warmer summers, or an increment in mean temperature.

Additionally, following the methodology proposed in [43], primary energy factors and greenhouse gas (GHG) emission coefficients were obtained based on the electricity mix of each country. Applying those factors to the energy demand, primary energy, and GHG emission savings were calculated. The annual savings obtained are presented in Table 3 and they relate to the operation phase after the retrofit. A cradle-to-cradle life cycle assessment is described in [28].

## 5. Conclusions

The aim of the present study was to investigate and optimize the energy performance of prefabricated geopolymer façade cladding panels made from large fractions of CDW. Firstly, a simulation model of the buildings was created to predict their energy performance. Secondly, a weather-normalized energy consumption was carried out for a like-for-like comparison between simulated and measured data, which led to the calibration of the building model matching simulation results and monthly utility bills. Thirdly, a scenario analysis was performed to test different configurations of the InnoWEE solutions to identify the retrofitting solution with the most positive impact. Finally, the environmental impact was analyzed for final energy, primary energy, and GHG emissions.

In order to compare existing and retrofitted scenarios, calibrated building models were simulated throughout the baseline (pre-retrofit) period and reporting (post-retrofit) period. The main outcomes from those simulations are listed below:

1. Energy savings achieved with InnoWEE passive solutions are in a similar range for the Greece and Italy demonstration sites.
2. ETICS-like panels are the most beneficial solution regarding energy savings for all locations.
3. Both ETICS-like and ventilated façade work more efficiently throughout winter months with higher heating demand.
4. The advantage of the ventilated façade throughout summertime might be underestimated since the model is based on ISO 6946:2017. This method does not consider the heat sink effect in the air cavity, which is the most beneficial feature of those systems for cooling purposes.
5. Cooling loads in warm climates might increase slightly by adding insulation, but such increases are negligible in the context of the full year consumption for the climates considered in this study.
6. For ETICS-like panels, reduced returns are obtained in annual energy savings beyond 8 cm of insulation thickness in the Greece demonstration site.
7. Insulation thickness is a decisive parameter to find an adequate balance between investment and operating cost.

Overall, this study has demonstrated that the InnoWEE ETICS-like panels and ventilated façades are capable of reducing the energy needs of a building, posing a feasible and potentially competitive alternative to be incorporated into the construction sector. In the future, the building models developed in this project can be used to simulate the performance of InnoWEE products in different building typologies and locations.

The outputs of the study suggest that circular economy can produce innovative materials and building elements, that can be competitive to similar market products if financially supported by public bodies. However, regarding the costs of the outer material, its inclusion into the initial investment would elevate the payback period and lower the feasibility for a market uptake. The reuse of materials and, therefore, the reduction on the impact of building retrofitting implicit to these materials indicate that policy makers and public stakeholders should support the use of these new materials with incentives and regulations.

As identified in this study, the thermal performance of the ventilated façade is limited and would be likely improved by the addition of thermal insulation between the existing wall and the ventilated cavity.

Regarding future research plans, the authors are already working on an assessment of highly insulated buildings for resilience to future climate changes. As it can be concluded by the presented work, highly insulated buildings may experience high cooling demands due to internal loads. From the resilience point of view, energy efficiency strategies must consider extreme events and warmer summers. The building models presented in this study will be exploited to understand the impact of climate change on buildings and to adapt retrofitting strategies accordingly.

## Figures and Tables

**Table 1:** ETICS (External Thermal Insulation Composite System)-like panel description. HDG: High-density geopolymer; EPS: Expanded polystyrene.

Features		Measurement (mm)
Height		400
Length		900
Width		78
Layering	HDG Layer	8
	EPS Insulation Layer 1	20
	EPS Insulation Layer 2	50
Thermal Resistance		2.037 (m^2^·K/W)

**Table 2:** Energy savings with InnoWEE passive solutions for all demonstration sites.

Italy, Padova				Romania, Bucharest			Greece, Voula		
Cfa: Humid Subtropical				Cfa: Humid Subtropical			Csa: Mediterranean Hot Summer		
TMY/Monitored				TMY/Monitored			TMY		
Reversible Heat Pump				Electric Heater/No Cooling Available			Reversible Heat Pump		
	Summer	Winter	Total	Summer	Winter	Total	Summer	Winter	Total
ETICS	−16.7%	37.4%	32%	-	4.40%	4.40%	−4%	49%	25.66%
VF	−1.6%	2.6%	2.4%	-	4.25%	4.25%	0.24%	4.74%	2.44%

Note: Classification taken from Köppen-Geiger Climate [42]. TMY: Typical Meteorological Years.

**Table 3:** Annual savings for Primary Energy and Greenhouse gas emissions with InnoWEE passive solutions for all demo sites.

Italy		Romania	Greece
Cfa: Humid Subtropical		Cfa: Humid Subtropical	Csa: Mediterranean Hot Summer
TMY/Monitored		TMY/Monitored	TMY
	Primary Energy Savings (kWh/year)		
ETICS	296.48	2867.12	1400.19
VF	37.47	2769.38	109.38
	GHG Emission Savings (kg/year)		
ETICS	62.99	545.70	427.11
VF	7.96	527.10	33.37

## Data Availability

Data available on request due to restrictions eg privacy or ethical. The data presented in this study are available on request from the corresponding author. The data are not publicly available due to confidentiality issues.
